# Is inappropriate secretion of anti-diuretic hormone (SIADH) the cause of hyponatremia in Legionella pneumonia?

**DOI:** 10.1186/cc10751

**Published:** 2012-03-20

**Authors:** P Schuetz, S Haubitz, B Mueller

**Affiliations:** 1 Medical University Clinic, Kantonsspital Aarau, Switzerland

## Introduction

Medical textbooks list Legionella as a differential diagnosis for the syndrome of inadequate anti-diuretic hormone (ADH) secretion (SIADH), but empirical evidence supporting this association is largely lacking. Partly this is explained by the analytical challenges of ADH measurement. With the recent availability of an immunoassay that measures the more stable ADH precursor peptide (CT-ProVasopressin), we sought to investigate whether increased ADH levels would explain hyponatremia found in Legionella patients.

## Methods

We measured CT-ProVasopressin and sodium levels in a prospective cohort of 925 pneumonia patients from a previous multicenter study with 31 patients having positive antigen tests for Legionella pneumophilia. We calculated Spearman rank correlations and multivariate regression models.

## Results

Legionella patients had higher rates of hyponatremia (sodium <130 mmol/l) (43% vs. 8%, *P *< 0.01), but similar median CT-ProVasopressin levels (pmol/l) (20 (12 to 26) vs. 26 (13 to 53), *P *= 0.89) compared to pneumonia of other etiology. In Legionella patients, high CT-ProVasopressin was not associated with low sodium levels, but showed a positive correlation with sodium levels (*r *= 0.42, *P *< 0.05). Independent of pneumonia etiology, CT-ProVasopressin were significantly correlated with the pneumonia severity index (*r *= 0.56, *P *< 0.05) and showed an association with risk for ICU admission (odds ratio per decile, 95% CI) (1.4, 1.2 to 1.6) and 30-day mortality (1.3, 1.2 1.4).

## Conclusion

We found no evidence that increased ADH secretion would explain low sodium levels in Legionella patients, or other pneumonia patients, challenging the common believe of Legionella causing SIADH. Rather, ADH precursors were upregulated as a response to severe disease. Future studies continuing to explore the cause of sodium disturbance in Legionella are warranted.

